# Knowledge, Attitude, and Practice of Blood Donation Among Undergraduate Medical Students in Azad Kashmir

**DOI:** 10.7759/cureus.7733

**Published:** 2020-04-19

**Authors:** Arslaan Javaeed, Rubina Kousar, Aalya Farooq, Saddaf Hina, Sanniya Khan Ghauri, Tayyaba Tabbasum

**Affiliations:** 1 Pathology, Poonch Medical College, Rawalakot, PAK; 2 Emergency Medicine, Shifa International Hospital, Islamabad, PAK; 3 Medicine, Combined Military Hospital (CMH) Rawalakot, Rawalakot, PAK

**Keywords:** blood donation, medical student, azad kashmir, pakistan, blood transfusion

## Abstract

Objective

To evaluate the knowledge and attitude of undergraduate medical students of Poonch Medical College about blood donation.

Methods

This cross-sectional study was done using a 27-item, validated, interviewer-administered questionnaire involving undergraduate medical students from March to October 2018. Informed consent and ethical clearance were secured.

Results

A total of 318 undergraduate medical students (response rate of 63.6%) was included in this study. Most respondents knew the difference between whole blood and blood components (294; 92.5%) and they also believed that spreading knowledge of blood donation among the health workers is a necessity (306; 96.2%). There was a statistically significant correlation between knowledge and attitude (p .021). Overall knowledge was higher among the female students (p = .019).

Conclusion

The study revealed an overall good level of knowledge and attitude among medical students. However, there are still areas of improvement such as blood donation and vaccination-related knowledge. The study also identified important facilitators and barriers to blood donation.

## Introduction

Safe and effective blood transfusion is a vital component in improving health care delivery and preventing the spread of blood-borne diseases worldwide. Every year, millions of lives are saved through blood transfusion, yet the quality and safety of blood transfusion are still of interest, especially in hospitals in developing countries [1-2]. According to the World Health Organization (WHO) recommendations, for any country to meet the minimum demand for blood, the donation should be at least 1% of the population [3]. It is estimated that around 234 million major operations are performed every year globally [4]. Blood scarcity is frequently encountered in hospitals and is due to an imbalance between the increasing demand for safe blood and blood products on the one hand and failure to organize regular blood supply due to misconceptions, perceived harms and risks, and a lack of motivation among potential donors [5]. Pakistan is among the countries where blood donation is still dependant mainly on the relatives of the patients and paid donors [6]. However, it is the goal of the WHO for all the countries to obtain all blood supplies from voluntary and unpaid donors by 2020 [7].

Medical college students can serve as a readily available pool of voluntary blood donors for the attached medical college hospitals and help reduce some of the scarcity of blood and blood products. Moreover, they can motivate a healthy population toward voluntary blood donation and thus may substantially narrow the gap between demand and supply of blood [8]. A medical student must have a high level of knowledge, positive attitude, and practice regarding blood donation that will motivate the society. This study was undertaken to assess the knowledge, attitude, and practice (KAP) of blood donation among undergraduate medical students of Poonch Medical College, Azad Kashmir, Pakistan. Similar studies were done in medical colleges of other regions of Pakistan but not in the Azad Kashmir region.

## Materials and methods

This was a cross-sectional, institution-based study carried out in Poonch Medical College in Rawalakot, Azad Kashmir, Pakistan. The study period was from May 2018 to December 2018. The interviewer-administered, 27-item questionnaire to assess blood-donation knowledge, attitude, and practice was developed by the researches and validated by epidemiologists. The questionnaire included 14 questions based on the respondent’s knowledge and 11 questions based on the respondent’s attitude toward blood donation. The question based on practice was divided into two themes: reasons behind blood donation and barriers behind not donating blood. The sample size was calculated assuming the prevalence rate of 50%, which revealed 384 required samples. In order to include the required samples, the study questionnaires were distributed to all five hundred students of either gender, pursuing Bachelor of Medicine, Bachelor of Surgery (MBBS) at Poonch Medical College. The total number of medical students at Poonch Medical College was five hundred. Out of them, students, 318 (response rate of 63.6%) completed the questionnaire and were included in this study. The research was carried out ethically, as stipulated in the Nuremberg Code, and informed consent was obtained from every participant of the study at enrollment.

Statistical analysis

Data were checked for completeness and errors were corrected. Descriptive statistics were used to present respondents’ knowledge of and attitude toward blood donation. Reasons and barriers to blood donation were presented in charts. The normality test was done using the Shapiro Wilk test and the Kolmogorov Smirnov test, which revealed non-normal distribution. Correlation between age, knowledge score, and attitude score was done by the Spearman correlation test. The relationship between knowledge and attitude and gender was observed by the Mann-Whitney U test. The relationship between knowledge and attitude and MBBS year was seen by the Kruskal Wallis H test. 

The analysis was performed in a 95% confidence interval using the Statistical Package for Social Science (SPSS), version 23.0 (IBM, Armonk, NY).

## Results

A total of 318 out of 500 (response rate of 63.6%) undergraduate (Year 1 to Year 5) medical students were included in this study. Among them, the majority were females (221; 69.5%), the median age of all respondents was 21 years, and the highest number of them were first-year MBBS students (116; 36.5%) (Table 1).

**Table 1:** Baseline characteristics of all respondents (n = 318) MBBS (Bachelor of Medicine, Bachelor of Surgery)

Characteristics	N (%)
Age in years	
Mean ± SD	21.22 ± 1.89
Median	21
Gender	
Male	97 (30.5)
Female	221 (69.5)
MBBS year	
Year 1	116 (36.5)
Year 2	42 (13.2)
Year 3	34 (10.7)
Year 4	61 (19.2)
Year 5	65 (20.4)

**Figure 1:**
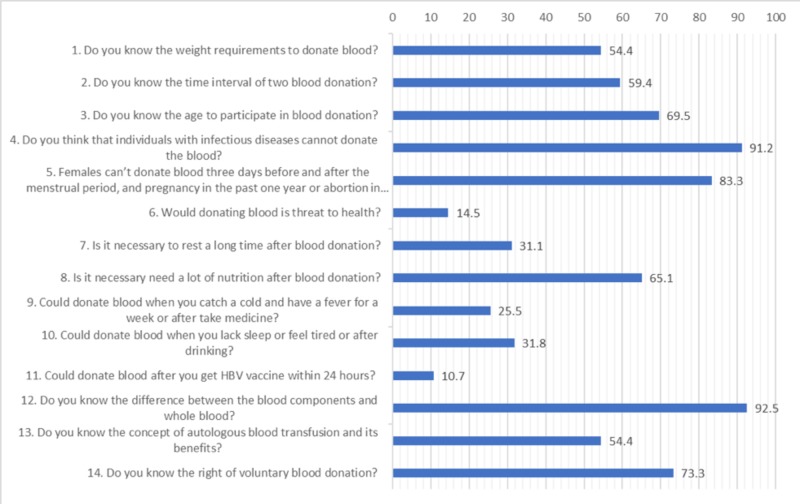
Respondents’ knowledge of blood donation (n = 318)

A total of 14 questions were asked to assess respondents’ knowledge of blood donation. The highest number of respondents gave the correct answer when asked if they know the difference between whole blood and blood components (294; 92.5%). In contrast, only 34 (10.7%) respondents knew that blood donation should not be done within 24 hours of the hepatitis B virus (HBV) vaccination. All knowledge-related questions were presented in Figure 1.

**Figure 2:**
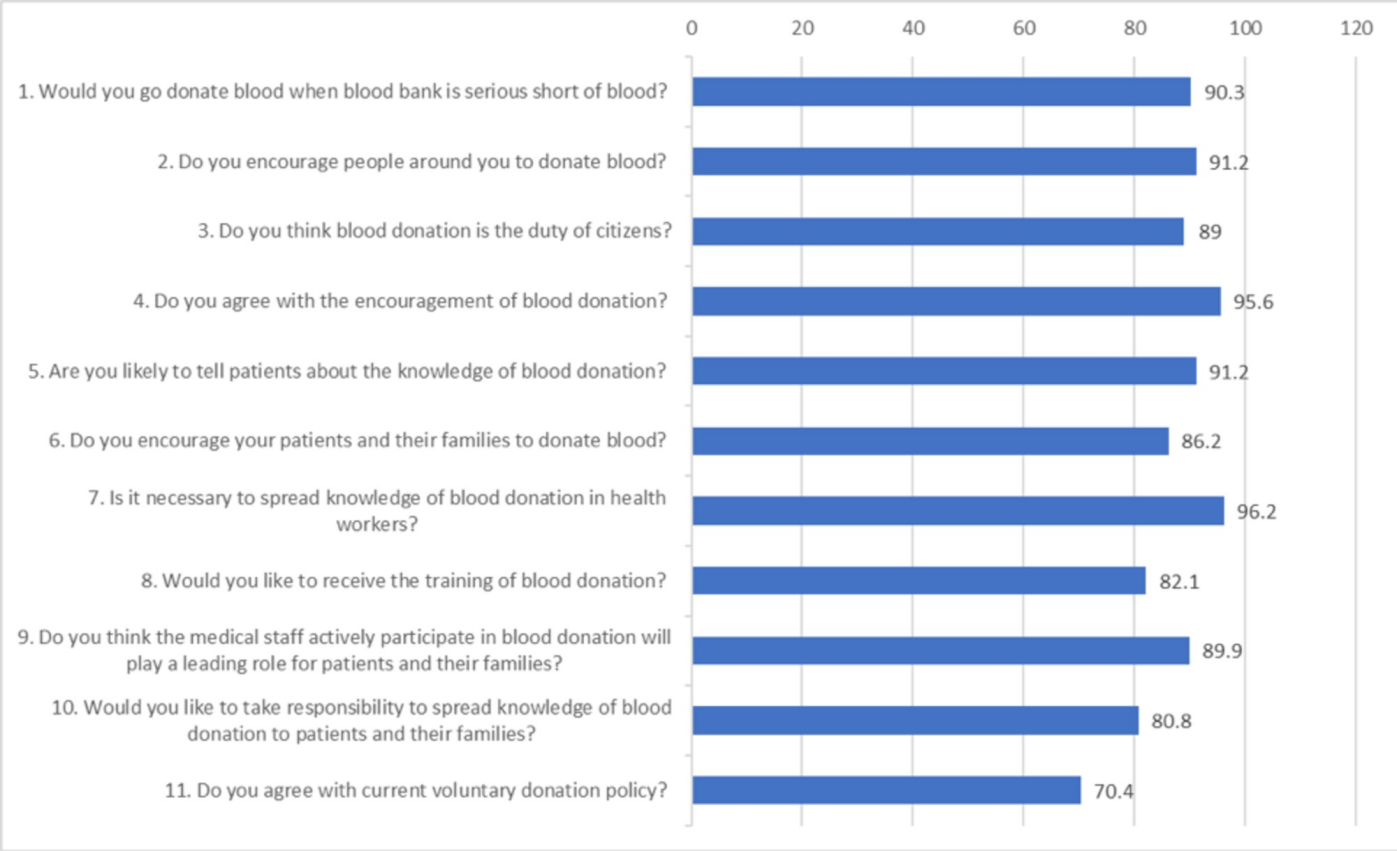
Respondents’ attitude toward blood donation (n = 318)

The clear majority believed that the spreading of knowledge of blood donation among health workers is a necessity, 306 (96.2%) whereas, relatively fewer, 224 (70.4%) respondents agreed with ‘current voluntary donation policy’. All other attitude-related questions were presented in Figure 2.

**Figure 3:**
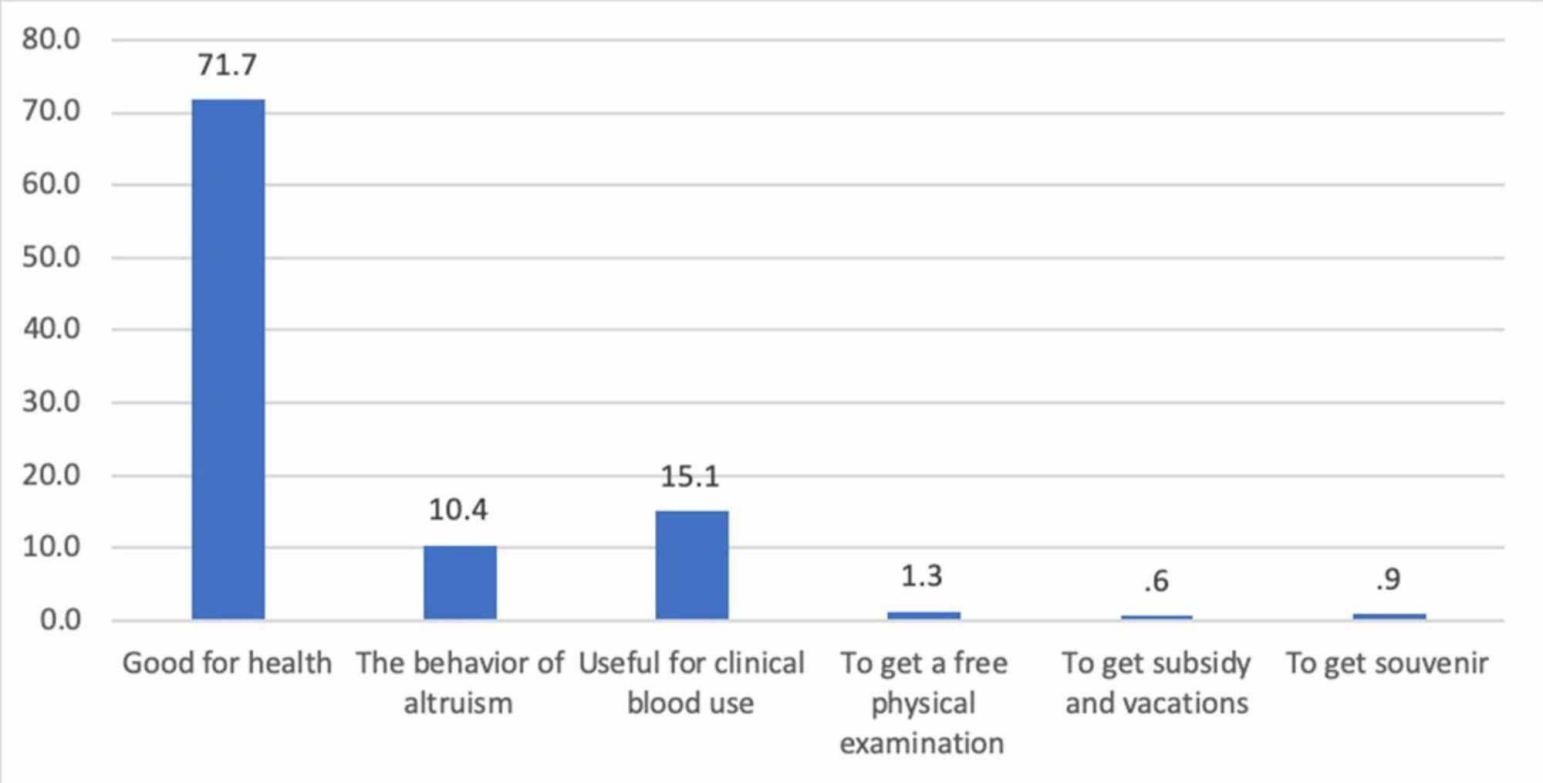
Distribution of all respondents by reasons for blood donation (n = 318)

**Figure 4:**
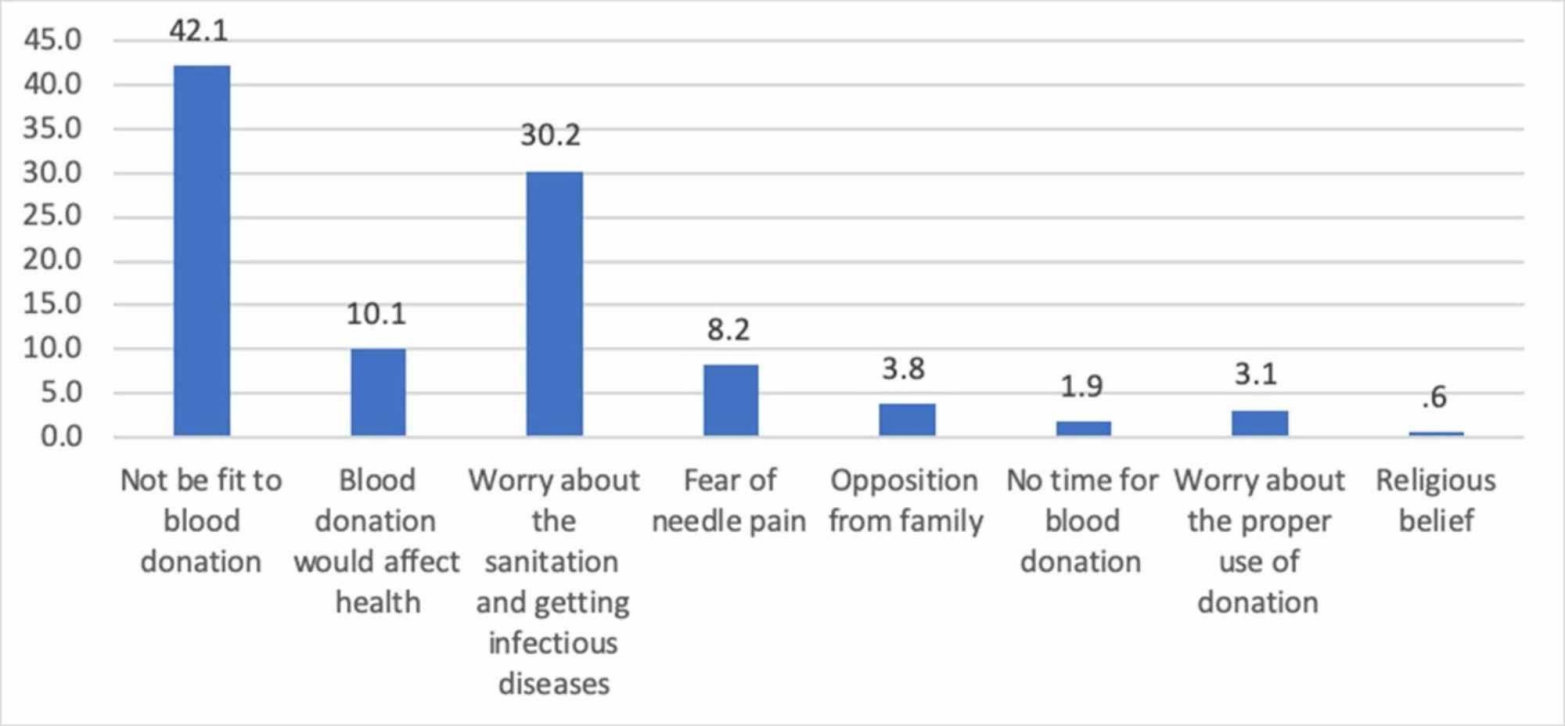
Distribution of all respondents by barriers to blood donation (n = 318)

The most important reason for blood donation was "good for health" according to 228 (71.7%) respondents and the most significant barrier was "not be fit to blood donation" thought by 134 (42.1%) respondents (Figures 3-4).

A statistically significant correlation was found between respondents’ age and attitude (p-value .015), years of study and attitude (p-value 0.006), and respondent’s knowledge and attitude (p-value .021) (Table 2). There was also a statistically significant relationship between knowledge and gender (p-value .019) with females having higher mean knowledge.

**Table 2:** Correlation between age, gender, years of study (MBBS), and knowledge with attitude (n = 318) Gender = Male or Female (p<0.05 taken as significant)* MBBS = Bachelor of Medicine, Bachelor of Surgery

Variables	Attitude p-value*
Age	.015
Gender	0.480
Years of Study (MBBS)	.006
Knowledge	.021

## Discussion

Blood transfusion is an indispensable component of global health care, which saves millions of lives every year. Every second, someone in the world needs blood for surgical or gynecological conditions [9]. All medical doctors should have sufficient knowledge about safe blood donation. Therefore, medical students have often been recruited for KAP studies about blood donation globally [5]. This study showed poor knowledge of some of the aspects of blood transfusion addressed by question nos. 6, 7, 9, 10, and 11. Ninety percent of the study population was aware of the high risk of transmissible disease through blood donation, which is critical in following safe blood donation practices. Although overall knowledge is not too poor as compared to some of the similar studies, this knowledge gap should be minimized [10-12]. 

The current study showed a very high percentage of positive answers to attitude-related questions. In contrast, a similar study was done in three medical colleges of Pakistan in 2012 only showed an overall 42% positive attitude regarding blood donation [13]. The Internet and easy availability of information may have played a role in improving the attitude of the medical students about blood donation. This study showed that the clear majority of the students, 90.3% will actually donate blood if there is a blood shortage in the blood bank, which is a very positive attitude. Most of the previously published studies did not reveal the blood donation willingness rate higher than the current study [14-16]. The most important barriers for blood donation was the lack of fitness and fear of getting an infection during the procedure. Similar barriers were observed in many developing countries [17-19]. A review study about the knowledge, attitude, and practices regarding blood donation in 17 developing countries, including Pakistan, stresses the need for creating awareness about voluntary blood donation based on country-specific, contextual knowledge, and attitudes [20]. It also maintains that fear is the most hindering factor behind donating blood. Mass media campaigns and safe methods for collecting donated blood can boost the confidence of people to make this a routine practice [21].

Surprisingly, personal health benefit was the primary reason cited for blood donation. As students from the first and second years comprised more than half of the studied population, this percentage could be attributed to the lack of an in-depth understanding of the critical need for blood donations in the country for clinical use. A study carried out amongst doctors, and paramedics in Pakistan highlighted a difference in their knowledge, which was reflected in their blood donation practices (76% of the doctors being blood donors as compared to 41% of paramedics) [22].

The most common barrier to blood donation was stated as a lack of fitness as perceived by the students. It is unexpected of a student population at a medical college to state such a response as a consensus but one plausible explanation could be that our study population comprised 70% of female participants and due to endemic anemia in adolescent girls (47.9%) in this region, it could be a reality [23]. However, this factor needs to be addressed in further studies.

One-third of the medical students had concerns about getting a blood-borne infection due to inadequate sterilization and unsafe blood collection practices, which was the second most common barrier in donating blood. A Pakistani study that documented that 67% of the medical students had never donated blood before shared similar concerns about getting infected during the process of blood donation [24]. Ensuring strict sterile conditions and a proper screening protocol during the collection process can mitigate the fears of the public.

The current study revealed a positive correlation between knowledge and attitude, which means if we can minimize the specific knowledge gaps, the attitude should also go towards a positive direction.

Limitations

The current study had several limitations, such as being a single-centered study, slightly less adequate sample size, unequal sample size from different medical years, and so on. Due to these limitations, the study findings may not be generalized.

## Conclusions

The study identified an overall good level of knowledge and attitude towards blood donation among the medical students in Azad Kashmir although knowledge was less than attitude. There was a positive association between knowledge and attitude levels, meaning increasing one will increase the other. In terms of practice, the study identified several facilitators and barriers to blood donations. This study encourages further studies on how to work on these facilitators and break the barriers.
